# Infrared Small Target Detection Method with Trajectory Correction Fuze Based on Infrared Image Sensor

**DOI:** 10.3390/s21134522

**Published:** 2021-07-01

**Authors:** Cong Zhang, Dongguang Li, Jiashuo Qi, Jingtao Liu, Yu Wang

**Affiliations:** Science and Technology on Electromechanical Dynamic Control Laboratory, Beijing Institute of Technology, Beijing 100081, China; zcbxl@bit.edu.cn (C.Z.); qjsphd@bit.edu.cn (J.Q.); jingtao_liu@bit.edu.cn (J.L.); yuwang@bit.edu.cn (Y.W.)

**Keywords:** trajectory correction fuze, infrared image sensor, small target detection, density-distance space

## Abstract

Due to the complexity of background and diversity of small targets, robust detection of infrared small targets for the trajectory correction fuze has become a challenge. To solve this problem, different from the traditional method, a state-of-the-art detection method based on density-distance space is proposed to apply to the trajectory correction fuze. First, parameters of the infrared image sensor on the fuze are calculated to set the boundary limitations for the target detection method. Second, the density-distance space method is proposed to detect the candidate targets. Finally, the adaptive pixel growth (APG) algorithm is used to suppress the clutter so as to detect the real targets. Three experiments, including equivalent detection, simulation and hardware-in-loop, were implemented to verify the effectiveness of this method. Results illustrated that the infrared image sensor on the fuze has a stable field of view under rotation of the projectile, and could clearly observe the infrared small target. The proposed method has superior anti-noise, different size target detection, multi-target detection and various clutter suppression capability. Compared with six novel algorithms, our algorithm shows a perfect detection performance and acceptable time consumption.

## 1. Introduction

Detecting infrared small targets on the ground at long distance is a crucial mission in many defense and anti-terrorist applications. Wheeled and tracked vehicles represent typical classes of small militant ground targets. Recently, the detecting sensor based on the trajectory correction fuze has received much attention [1]. In previous research, the traditional detecting method could not effectively detect targets in the complex environment [2]. Therefore, this paper mainly aims to improve the detection accuracy of infrared small targets on the trajectory correction fuze.

Among various detecting methods, thermal infrared imaging is a passive model to effectively detect small targets at long distance. Compared with the visible image sensor, radar [3] and laser [4], the infrared image sensor has capabilities of strong anti-interference, good concealment and all-day work. At present, the forward-looking infrared sensor (FLIR) adopts the superior microbolometer with the noise equivalent temperature difference (NETD) of 50 Mk, the pixel pitch of 12 μm and wavelength of 3–14 μm. In previous research, the authors of [5] designed a correction fuze and reserved space for an infrared image sensor. However, the important parameters of the sensor were not calculated. We aim to make a graceful integration of the infrared image sensor and the fuze that benefits the target detection.

The trajectory correction fuze could reduce the circular error probable (CEP) by adding the correction function to the mortar without any modification of the projectile. Generally, the onboard computer calculates the correction value by the detected target’s azimuth. Then, the original terminal trajectory is changed by a pair of rudders which could produce the aerodynamics. Existing research on the correction fuze combined with the infrared image sensor is still in the initial stage. For example, the authors of [6,7] researched the influences of the velocity, rotation rate and pitch on the dynamic response during terminal correction. However, the target detection method was ignored when the aerodynamic feature and correction strategy were analyzed. Therefore, accurately detecting the infrared small target is essential for realizing the fuze’s function [8].

The infrared small target has three unique characteristics, such as small physical dimensions, and low thermal and visible signatures [9]. Generally, small target size does not exceed 0.15% of the image resolution. Likewise, without dissipating any thermal energy, the vehicle body itself only reflects some incident energy, which is almost equal to the energy reflection of the surrounding environment. Most of the energy difference is generated by the motor, and its infrared band is basically in the range of the long wave (8–14 μm). Such low thermal energy produces a minuscule signal in the infrared image sensor. As the detection distance increases, the signal-to-noise ratio (SNR) of the target degrades drastically. Therefore, these challenges make infrared small target detection an unusually difficult task.

Existing traditional infrared small target detection approaches mainly include the use of spatial-only information in individual single frames and the spatial-temporal information in image sequences, where the spatial-only method could be divided into two categories: filter-based methods and human vision system (HVS) methods. The former designed filters to express noise according to the statistics of local pixels, such as max-median [10], max-mean [11], and morphological [12] transformation. Although these typical filters are of lightweight calculation, they are extremely interfered with by noise. Correspondingly, the improved filter methods of median [13,14], mean [15] and Gaussian [16] were proposed to effectively suppress clutters. In addition, some improved morphological methods such as Top-Hat [17] and region grow [18] were studied. However, the initial elements selected by these methods should be highly matched with various clutter shapes, otherwise the performance will be degraded. To summarize, these methods partly improve the robustness, but still have a high false detection rate with complex background and variable target size.

On the contrary, the HVS [19] methods are directly applied to the target itself. The local contrast method (LCM) [20,21], simulating the attention mechanism of the HVS, is often used to detect targets. Later, a series of improved algorithms based on this were proposed, such as improved LCM (ILCM) [22], novel LCM (NLCM) [23] and relative LCM (RLCM) [24]. By redefining the contrast parameters of the central region, the pixel-sized noises with high brightness (PNHB) are suppressed and the target is enhanced. Moreover, other methods optimize the rectangle structure of LCM to detect targets with different sizes, such as double-neighborhood (DLCM) [25], tri-layer (TLCM) [26], multiscale patch-based (MPCM) [27] and high-boost-based multiscale (HBMLCM) [28]. Although local contrast is further enhanced, sparse clutter elements are also highlighted [29]. Besides, the above methods are cyclically and complexly calculated pixel-by-pixel, which leads to high computational costs and cannot meet the real-time requirements of target detection.

Recently, convolutional neural networks (CNN) [30,31] have been widely used in target detection and recognition. Derived methods consist of one-stage methods, such as single-shot multi-box detector (SSD) [32] and you only look once (YOLO v1-v5) [33,34], and two-stage methods such as Fast-RCNN [35] and Faster RCNN [36], providing a new backbone, activation function, loss function, etc. These methods all require a large number of datasets for training to obtain model parameters. However, the fuzes often lack prior knowledge and cannot obtain huge datasets, and the size of the target is too small to extract features.

As described, our main goal in this paper is to effectively detect small infrared targets in the application of the trajectory correction fuze, and experimentally demonstrate that our method could deal with the challenges associated with long distance, small variable size and minimal thermal signature in a real infrared scene. The comprehensive performance is better than the existing methods. Our main contributions include:

(1) On the basis of [5], the main parameters of the infrared image sensor were selected, and then the correctness was verified through outdoor experiments.
(2) Inspired by filtering methods, we proposed a novel two-dimensional density-distance space to obtain the density peak pixels by full use of image information.
(3) A new pixel growth method was presented to effectively suppress clutters. Then, the real targets were selected from the density peak pixels.
(4) Three experiments proved the robustness and effectiveness of the algorithm and the applicability of the trajectory correction fuze. Especially, our method maintained a good detection performance without increasing the processing time compared with the previous existing methods.

## 2. Application Background

The infrared image sensor is located on the front of the fuze. Its detection ability is the premise for completing the correction function. Therefore, the parameters of the infrared image sensor should be calculated based on the trajectory feature of the mortar.

### 2.1. Design of the Fuze 

In this paper, the fuze is used in the tail-stabilized mortar with three characteristics. First, the projectile rotates at a speed of no more than 2 r/s. Second, the pitch and yaw angle of the projectile changes at a high frequency because of the curvature of the trajectory. The above problems lead to the frequent changes of the image background. Finally, the conventional terminal trajectory distance is 1.5 km. With the mortar flying at an initial speed of 272 m/s, the total time is within 8 s. The rest of the time for target detection is extremely short, excluding the correction and solution time. Therefore, the number and function of sensors should be balanced due to the limitation of response time and the space size of the fuze.

**Figure 1:**
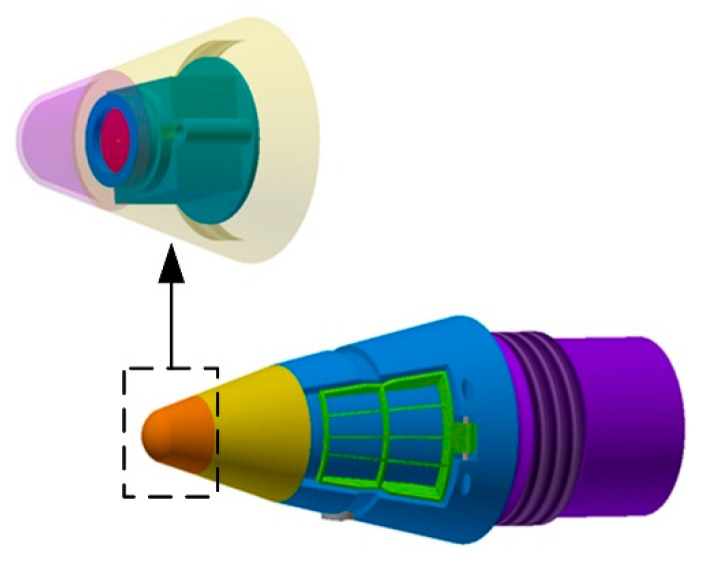
Model of the trajectory correction fuze and the infrared image sensor.

As shown in Figure 1, the organization is composed of three parts. The aft part, shown in purple, connects the fuze and the projectile with threads. The mid part, indicated by blue, is used to externally fix the rudder blade and internally contain the sensors for control. When the mortar rotates, the fuze can rotate in the opposite direction to the projectile to ensure a stable field of view for the image sensor. The front part, equipped with the infrared image sensor, is used for navigation. In addition, the detail of the infrared image sensor is shown at the top of Figure 1, where the fairing is at the top. The green base is used to fix the sensor and lens. Therefore, the infrared image sensor is not affected by the rotation of the projectile due to the reverse function of this structure. Correspondingly, this design makes the field of view stable and ensures the detection capability.

### 2.2. Connection between Infrared Image Sensor and Fuze

When the mortar flight is in the terminal trajectory, the parameters of the trajectory and the infrared image sensor will affect the detection effect directly. Therefore, the detection algorithm should be based on the workflow of the correction fuze and the infrared image sensor. Table 1 lists the trajectory parameters of conventional mortar and parameters of the infrared image sensor selected in this paper. 

According to the trajectory equation of mortars, the terminal flight time is generally within 8 s when the detection distance is 1500 m and the launch angle is 53°. Obviously, this time is sufficient for algorithms with high FPS. However, most of the time is spent on the onboard computer calculation and trajectory correction, leaving a short amount of time for target detection. Simultaneously, at the beginning of the terminal trajectory, the pitch angle of the projectile changes rapidly within the integration time. This causes the change of the background on the field of view. Therefore, the classic frame difference method using time domain should not be applied. The appropriate method is to detect the target in a single image.

Notably, in this paper, the parameters of the infrared image sensor were selected based on the target’s size, under the premise that the temperature difference between the target and background can be detected. Due to the fact that the detection range of most current uncooled staring VOx microbolometers is far greater than 1500 m, the infrared small target is clearly visible on the field of view on the terminal trajectory. However, unlike missiles, overload caused by mortars’ launching phase will damage the lens assembly. Therefore, a stable coaxial spherical lens assembly is used. The transformation relation between the infrared image sensor and the target is calculated as follows:

(1) $$\frac{R}{f}=\frac{H}{h}$$

(2) $$Fov_{col}=2\arctan\left(\frac{nd}{2f}\right)$$

(3) $$Fov_{row}=2\arctan\left(\frac{md}{2f}\right)$$

where *f* is the focal length of the infrared imager, *R* and *H* are the detection distance and size of the target and *h* is the pixel size of the detectable target on the pixel array. The target size is set to 1-line pairs according to the detection degree of the Johnson Criteria. The long-wave sensor with the spectral band 7.5–13.5 μm is selected, because the thermal wavelength emitted by the target is about 8–14 μm. *Fov_col_* and *Fov_row_* are the vertical and horizontal field of view, respectively. *n*, *m* and *d* represent the array format and single pixel size. These parameters are used to calculate the equivalent detection experiment of the infrared image sensor.

Generally speaking, the fuze needs to detect multi-targets with different sizes. Moreover, due to the long detection distance, the target usually contains only a few pixels and does not exceed 0.15% of the size of the image. Therefore, an infrared small target detection algorithm based on a single image is proposed. The algorithm should not be affected by target size and background.

## 3. Density-Distance Space

**Figure 2:**
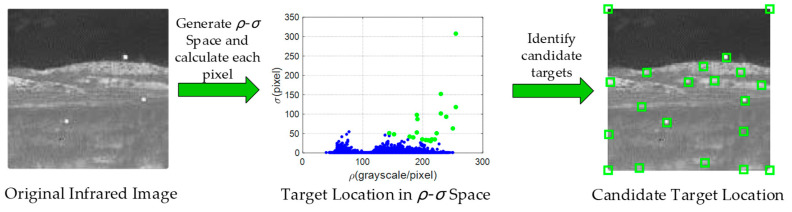
Flowchart of the density-distance space method.

The discussion in Section 2 shows that the algorithm should have three features, namely adaptive infrared small target detection capability, scales’ changeable adaptability and low running time. In an infrared scene, most small targets exist in relatively open areas and have higher grayscale than their surroundings. Correspondingly, infrared small targets should have two features, maximum grayscale in the local region and farther distance from any higher gray value pixel. Figure 2 shows the flowchart of the density-distance space for the infrared small target detection method. First, the two main parameters density-*ρ* and distance-*σ* are generated from the original infrared image. Then, the *ρ* and *σ* values of all pixels are converted into a special two-dimensional *ρ-σ* space. The green candidate targets are selected from this space through an adaptive threshold, including real multiple targets and false targets. 

### 3.1. Definition of Density and Distance

Infrared small targets usually have high gray value, lower pixel number, certain contrast or other features. This can be summarized as having a higher gray value in a limited area compared to neighboring areas. According to this definition, infrared small targets can be regarded as having a higher “density”. In detail, the gray value is regarded as “quality”, and the number of pixels is the “volume”. Correspondingly, a simple but effective method to define the density of each pixel is shown in Equation (4):

(4) $$\rho \left(i,j\right)=\frac{1}{n}\sum_{\left(i,j\right)\in \varepsilon }G\left(i,j\right)$$

where *ρ*(*i*,*j*) is the density of the pixel at the (*i*,*j*) position. *G*(*i*,*j*) is the gray value, and *n* represents the number of pixels in the area of *ε*. The density value reflects the average gray features of the target and concentrates features on one pixel. Furthermore, by detecting a single pixel, the size and shape of the target will not affect the detection result.

**Figure 3:**
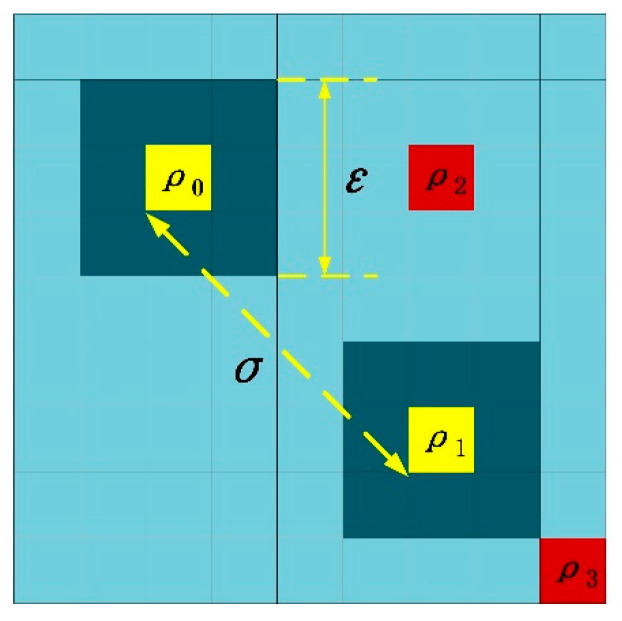
Calculation process of density and distance of pixels.

The parameter *σ* is defined as the distance feature of the pixel, which means the minimum distance between pixel *i* and any other pixel *j* with higher density. Equations (5) and (6) show the calculation of distance, where *d_ij_* represents the Euclidean distance between pixel-*i* and pixel-*j*, and *x* and *y* are the coordinate values of the pixels. Figure 3 shows the calculation process of density and distance. The area with light blue is the original image, and the deep blue is *ε*, which has a 3 × 3 size. The order of density is *ρ*_2_ < *ρ*_0_ < *ρ*_1_, *ρ*_3_. For pixel-*ρ*_0_, *σ* is the distance between *ρ*_0_ and *ρ*_1_, which is shown in yellow. Although *ρ*_2_ has a shorter distance, its density is less than *ρ*_0_. Correspondingly, the density of *ρ*_3_ is higher than *ρ*_0_, but the distance is longer than *ρ*_1_. Therefore, the two red pixels could not be selected as the correct distance value. It should be noted that when the pixel-*i* has the maximum density, there is no *σ*. We temporarily take its *σ* to the maximum value among all *d_ij_* values, because it may be the true target and cannot be ignored. Then, it will be compared with other pixels through the subsequent adaptive threshold.

(5) $$\sigma_{i}=\underset{j:\rho_{j}>\rho_{i}}{\min}\left(d_{ij}\right)$$

(6) $$d_{ij}=\sqrt{{\left(x_{i}-x_{j}\right)}^{2}+{\left(y_{i}-y_{j}\right)}^{2}}$$

### 3.2. Density-Distance Space

If a pixel has a maximum *ρ*-density in the local region and a long *σ*-distance at the same time, we call it “density peak”. Obviously, the small infrared target meets these features. Generally, pixels in a continuous background have low grayscale and are close to pixels with higher grayscale. Correspondingly, the number of density peaks is small. Therefore, a certain number of density peaks could be directly selected as candidate targets. This brings two benefits. One is that the detection accuracy will not be affected by target size and shape. The other is that the detection difficulty is reduced. Therefore, evaluating the density peak by two-dimensional *ρ*-*σ* space and adaptive threshold α, we select a certain number of candidate targets. The calculation of *α* is shown in Equation (7):

(7) α=ρ×σ

where *α* is mainly used to evaluate whether the pixel is a density peak. By multiplying *ρ* and *σ*, the difference of *α* between the real target and the background is enlarged. Then, all the pixels are sorted by *α*. The number of real targets is *n*. To ensure that the real targets will not be lost within the minimum calculation amount, we take at least 4*n* as candidate targets from density peaks with a larger *α*. Notably, it is almost impossible that a real target has two pixels with the same density, and the *σ*-distance of the lower-density pixel is extremely small. Therefore, the target region only has one density peak.

**Figure 4:**
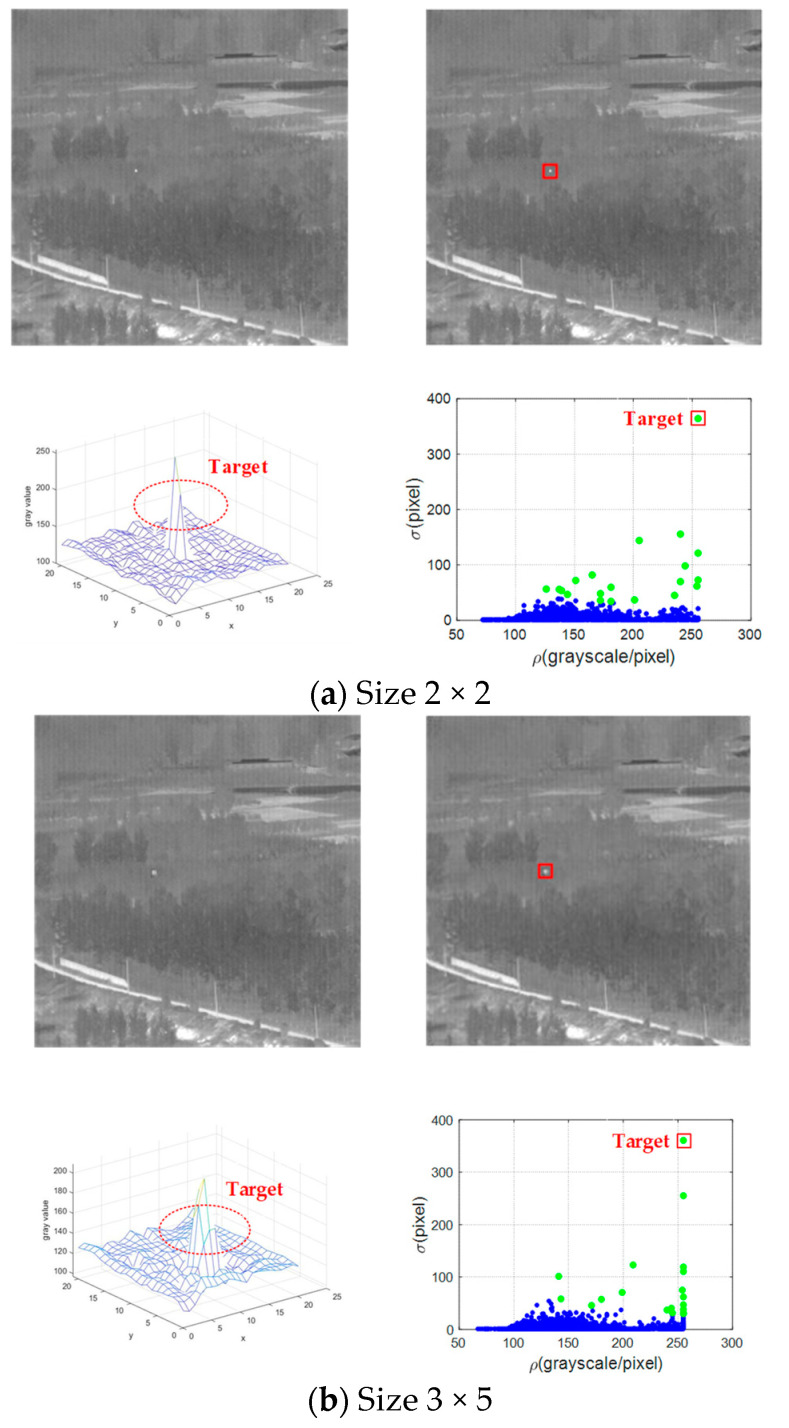
Detection results of the two targets with different size in the same condition. Both (**a**) and (**b**) contain 4 images, which are the original image, detection result, target region mesh and *ρ*-*σ* space, respectively.

Figure 4 shows the establishment and preliminary detection of *ρ*-*σ* space of two targets with different sizes at the same position. The sizes of the two targets in Figure 4a and b are 2 × 2 and 3 × 5, respectively. We extracted a region with 25 × 25 pixels containing the target. As shown, even if the target sizes are different in the original image, the targets with the same position have similar positions in the *ρ*-*σ* space, approximately. This test shows that the *ρ*-*σ* space has the ability to detect multi-scale targets.

### 3.3. Adaptive Pixel Growth

Notably, it is easy to confuse the detection of real targets, where some density peaks are located at the edges of some background regions. Therefore, an adaptive pixel growth (APG) algorithm is used to eliminate false targets so as to retain the real targets.

The APG method is similar to that of region growth. Each candidate target (pixel) is regarded as a “seed”, and represented by *T_k_*(*x*,*y*), where *k* is the number of candidate targets and (*x*,*y*) is the coordinate of the pixel. The seed grows in the direction of its eight neighboring connected pixels. The growth condition depends on the grayscale difference between the seed and its neighboring pixels. When the difference is less than the threshold, the seed grows one step, otherwise it stops growing. We use *Th* to denote the adaptive threshold to limit the growth of the seed, as shown in Equations (8) and (9):

(8) $$Th=\eta \times \left(G_{T_{k}}-{\bar{G}}_{P_{k}}\right)$$

(9) $$P_{k}\left(i,j\right)=\left\{\left(i,j\right)|x-m\leq i\leq x+m,y-n\leq j\leq y+n,m^{2}+n^{2}\neq 0\right\}$$

where *P_k_* denote the pixels around the seed *T_k_*, *G_Tk_* and *G_Pk_* represent the grayscale of the seed *T_k_* and pixel *P_k_* and ${\bar{G}}_{P_{k}}$ is the average gray value of the pixels *P_k_*(*i*,*j*). The range of *P_k_* is the pixels in a certain area around but not containing the seed, as shown in Equation (9). *m* and *n* are positive integers, *η* is the threshold coefficient and the range is 0–1, which is obtained by large numbers of experiments. After one step of pixel growth, the seed, *T_k_*, extends to one or more pixels in the set *P_k_*. Equally, the pixels in the set *P_k_* which meet the threshold, *Th*, become new seeds. All seeds follow this strategy in each step of the algorithm. When the growth of seeds cannot meet the threshold, *Th*, the growth stops. The total number of pixels after the final growth of each seed is regarded as area *S_k_*. 

**Figure 5:**
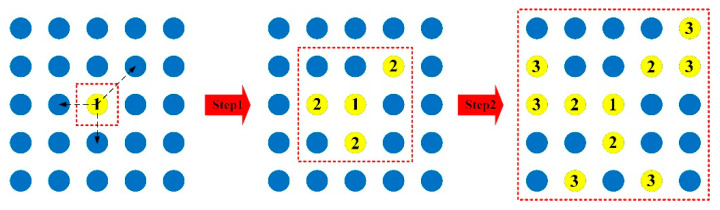
Process of the APG method. From left to right is the two-step growth process of the No. 1 seed.

Figure 5 shows the process of APG, where seed, *T_k_*, is shown in yellow, and the surrounding pixels, *P_k_*, are shown in blue. The red dotted frame indicates the range after each step of APG. At the beginning, there are three pixels around the seed, *T*_1_(*x*,*y*), within the growth threshold, *Th*, which are *P*_1_(*x* + 1,*y* + 1), *P*_1_(*x*,*y* − 1) and *P*_1_(*x* − 1,*y*), respectively. After the first step, three new seeds marked with number 2 are added. Similarly, after the second step, the new seeds are marked with number 3. For this seed, the area *S*_1_ contains 10 pixels after two steps of APG.

**Figure 6:**
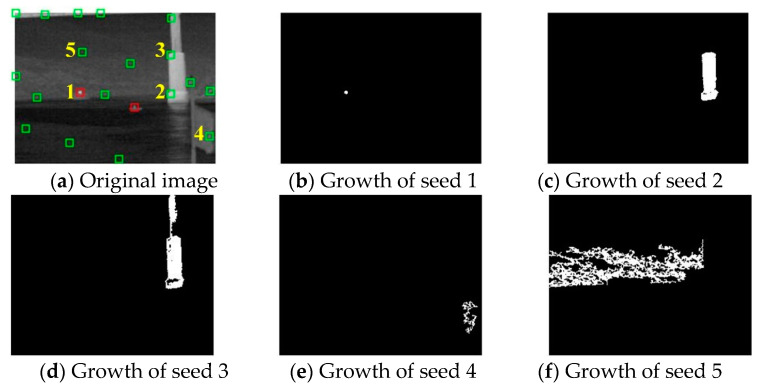
Growth features of seeds: (**a**) contains 5 seeds in different regions, (**b**) is the seed of the target region and (**c**–**f**) are the seeds in clutter regions.

The growth feature of seeds in the target region, edge region and clutter region is shown in Figure 6, and 20 candidate targets including 2 real targets are detected in Figure 6a. The seed 1 is in the target region, and the number of growth steps is small, as shown in Figure 6b. Correspondingly, the area *S_k_* of the seed is small. Figure 6c,d show the growth results of seeds 2 and 3. For a seed in the edge region, such as buildings, pixel set *T_k_* will grow along the high grayscale edge under the premise of not exceeding *Th*. Therefore, the entire edge region will be covered by the area of seed growth. Especially, when the seed is located at the junction of two connecting regions, the growth will contain both regions. Obviously, the growth areas of seeds in the edge region have high density. Figure 6e,f show that the growth areas of seeds 4 and 5 are located in clutter regions, such as clouds, sea–sky line and other float backgrounds. Clearly, the growth areas of clutters are sparse. Through the comparison of the above three conditions, the target region has the smallest area *S_k_*. Therefore, the APG method could effectively remove the clutters and background, and detect real targets.

## 4. Experimental and Analysis

In this section, the parameters of the infrared image sensor are first verified to ensure that the correction fuze can clearly capture infrared targets. Then, our proposed algorithm is tested to verify its effectiveness, robustness and real-time performance by simulation and the hardware-in-the-loop experiment. There are two key parameters that should be set in the following experiments. First, *n* is set to 5 due to the fact that the number of targets we detected generally did not exceed 5. Then, for a small target with several pixels, the growth directions of its neighboring pixels surrounding the seed are usually 3–5, which occupy 40–60% of the 8 directions. Therefore, *η* is set to 0.4 to limit the growth area of real targets. We do not need any further parameter changes in all of the following experiments.

### 4.1. Equivalent Experiment about Detection Capability of the Infrared Image Sensor 

The prerequisite for the algorithm verification was that the infrared imager could clearly observe infrared small targets. For this purpose, both equivalent size and temperature difference should be considered. We used FLIR’s Tau2 infrared imager with the same parameters as in Table 1 for experiments. A vehicle model connecting with an electrically conductive board was used to replace the infrared target. A temperature sensor powered by a battery was connected with the electrically conductive board through its own thermometer. When the surrounding environment temperature, *T*_0_, is known, the temperature sensor could control the temperature, *T*_1_, of the electrically conductive board to maintain the temperature difference (*T*_1_ − *T*_0_). The infrared image sensor was carried by an UAV to achieve the long-distance detection. The experimental conditions are shown in Table 2.

The size feature of the tank was 2.3 m according to the data, and the size of the infrared target model was 0.1 m. Correspondingly, when the real detection distance is 1500 m, the equivalent distance should not be less than 65 m. Similarly, the temperature difference should also be equivalent, but it is not linear with the detection distance. It needs to be multiplied by an atmospheric transmittance coefficient, *τ_a_*. In Reference [37], the range of *τ_a_* is 0.3–0.7 under the bad condition of cloud cover. Therefore, *τ_a_* is set to 0.5, based on the average. For the ground environment, when the temperature difference between the vehicle target and the surrounding environment is 15 K on average, the equivalent temperature difference is 7 K.

**Figure 7:**
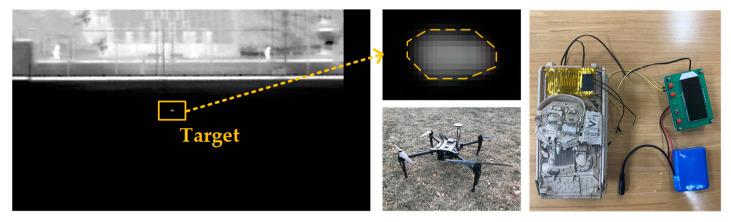
Experiment of detection capability of the infrared imager. The target observation result is shown on the left. The equipment is shown on the right.

The experimental equipment, arrangement and detection results are expressed in Figure 7. The highlighted region in the image is the small infrared target. Notably, the target is vignetted due to the diffusion of its own thermal radiation, leading to the changes of the target’s shape and size. However, the density-space method will not be affected. We only detected one pixel with the highest density in the target region. The experiment proved that the infrared image sensor could clearly observe the target at long distance, and simultaneously provided a guarantee for subsequent simulation experiments.

### 4.2. Simulation of Algorithms

Simulation experiments were mainly used to prove the effectiveness of the proposed algorithm. Notably, the simulation was run on a laptop with a 2.8 GHz Intel i7-7700HQCPU processor and 8 GB RAM. In order to evaluate our algorithm objectively, only one key parameter should be preset in this simulation: the number of candidate targets (seeds). According to the analysis in Section 3, we set the number of candidate targets to 20 so that the infrared image can contain no more than 5 real targets.

**Figure 8:**
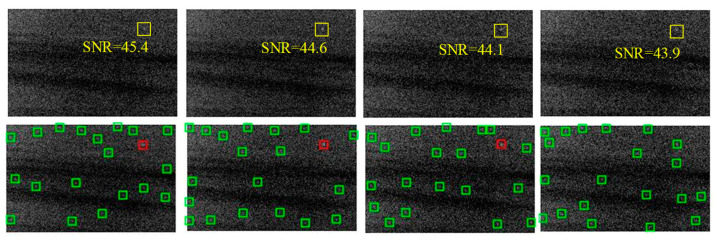
Simulation of the anti-noise ability of the algorithm. The SNR decreases from left to right.

The detection result is easily affected by noise. Therefore, the anti-noise ability should be tested. In order to reflect the effect intuitively, an image with one infrared target was selected, and different levels of Gaussian noise were added in this picture, respectively. SNR is defined as in Equation (10), where $\sigma_{t}^{2}$ is the grayscale variance of the target region and $\sigma_{n}^{2}$ is the variance of Gaussian noise. Figure 8 shows the noise with different SNR and target detection results. Obviously, the target is hard to detect as the SNR decreases gradually. When SNR was from 44.1 to 45.4, we obtained a perfect detection result. The target missed when SNR was reduced to 43.9. The changes of position of the remaining candidate targets are caused by the randomness of Gaussian noise. Consequently, the lower limit of SNR that the algorithm could detect the target at is around 44. When SNR was higher than 44, the algorithm could detect the real target. Otherwise, depending on the distribution of noise, it may not detect the real target. The test clearly showed a certain degree of anti-noise ability of the algorithm based on density-distance space and the APG method.

(10) $$\mathrm{SNR}=10\mathrm{lg}\frac{\sigma_{t}^{2}}{\sigma_{n}^{2}}$$

**Figure 9:**
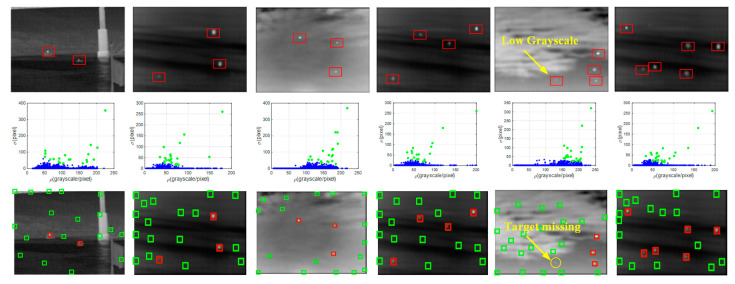
Simulation of the detection ability of multi-target for six images. The first row is the original images, the second row is the corresponding density-distance space and the bottom row is the detection results.

In addition, the multi-target detection ability of the algorithm in different environments was tested by simulation. We chose six typical images with different backgrounds, target number and target size to accurately test the robustness of the algorithm. Figure 9 shows the *ρ*-*σ* space and the detection results. The targets are marked with red rectangles. Two pairs of ground and sky images, respectively containing 3 and 4 infrared targets with different size, grayscale and regions, were selected. Besides, the limit of the detection ability of the algorithm was tested by the last image with 6 targets. All backgrounds in the image sequences include clutters with different types and shapes. The details of the original images are listed in Table 3.

In Figure 9, the red rectangles represent the real targets, and the green ones represent the candidate targets. In the first four images, all real targets were well-detected under the interference of various clutters. No false detections or missed detections occurred. Two extreme conditions should be considered. In the fifth image, only three of the four real targets were detected because the missing target has a minuscule grayscale. In the sixth image, all targets were detected by setting *n* to 6 individually. The results proved that our method had a superior robustness, adaptability and efficiency for the detection of multiple infrared targets in most conditions. However, the target with a low *α* value will still be missed when its thermal signature is lower than clutters.

To further evaluate the comprehensive effectiveness of the proposed algorithm, six novel algorithms were used for comparison, namely the Top-Hat method [17], the least squares support vector machine method (LS-SVM) [38], the high-boost-based multiscale local contrast measure (HBMLCM) [28], the multiscale relative local contrast measure (RLCM) [24], the multiscale patch-based contrast measure (MPCM) [27] and the minimum local Laplace of Gaussian (MinLocalLoG) [13]. We used the receiver operator characteristic (ROC) curve to compare the effects of the algorithm. The ROC is calculated by the true positive rate (TPR) and false positive rate (FPR), as shown below:

(11) $$\mathrm{TPR}=\frac{\mathrm{True}\;\mathrm{Positive}}{\mathrm{True}\;\mathrm{Positive}+\mathrm{False}\;\mathrm{Negative}}$$

(12) $$\mathrm{FPR}=\frac{\mathrm{False}\;\mathrm{Positive}}{\mathrm{Fasle}\;\mathrm{Positive}+\mathrm{True}\;\mathrm{Negative}}$$

The ROC curve is composed of FPR as the abscissa and TPR as the ordinate so as to provide a quantitative comparison of the detection performances. Meanwhile, the area under the curve (AUC) was the area included by the ROC curve and the axis of abscissa and ordinate. We prepared five different datasets of 200 frames for evaluation. Every frame has only one true target with a corresponding coordinate. In order to be considered a correct result, the detected target must have the same coordinate as that of the real target. The detection rate could be regarded as the AUC value, which means the proportion of the number of real targets detected in 200 frames. Meanwhile, the higher AUC value presents the better performance of our method to detect the real target in one frame.

**Figure 10:**
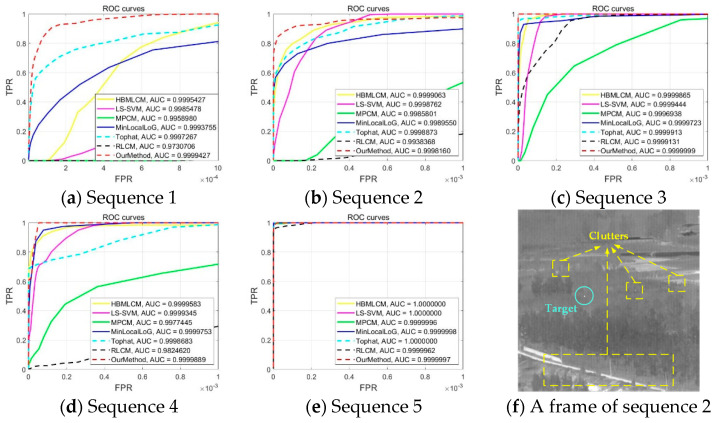
Simulation of the ROC curves of different algorithms and corresponding AUC values: (**a**–**e**) are the comparison results of 5 different sequences, where each sequence has 200 frames, and (**f**) is any frame of image in sequence 2, which is mainly used to specifically explain the reason for the low AUC value of our method.

Figure 10 shows the ROC curves and AUC value. The red dotted line represents the proposed algorithm. It can be seen from all ROC curves that the proposed algorithm generally has a superior detection accuracy. In sequences 3–5, the AUC value of our algorithm is almost close to 1. However, the detection accuracy of sequence 2 is not ideal. This is due to more clutter and the low target grayscale. For example, the second sequence shown in Figure 10f has many clutters with a small area, which seriously interfere with the step of pixel growth. The AUC value of our method was 6.02 × 10^−5^ and 9.03 × 10^−5^ lower than that of HBMLCM and LS-SVM. Thus, the detection rate of our method is slightly worse than that of LS-SVM and HBMLCM but better than the other four algorithms.

Then, the running time test was performed. We compared the time consumption of all algorithms by calculating the average time of 200 frames for each sequence, as listed in Table 4. Although the running time of our algorithm was slower than the other four algorithms, it was not more than an order of magnitude and had better performance. The total average running time is listed at the bottom of the table. The running time of the proposed method is almost equal to that of MPCM. Compared with MinLocalLoG, LS_SVM, HBMLCM and MPCM, our running time was more, by 0.0057, 0.0096, 0.0031 and 0.0006 s, but was still in the same order of magnitudes. Top-Hat had the shortest running time, which is about 3 times lower than the proposed algorithm. RLCM has the largest amount of calculation, surpassing most methods by 2 orders of magnitude.

The comprehensive performance and running time of HBMLCM were better, but its adaptability of clutter is weaker than our algorithm. However, the target detection task of the fuze was to firstly ensure the detection accuracy, and then improve the real-time performance as much as possible. Therefore, compared with other algorithms, our method was more applicable for fuze.

To summarize, our algorithm had a better capability and robustness than the other algorithms, and the running time was also acceptable. Compared with the six existing methods, the proposed method has the best performance of detection accuracy. Although Top-Hat has the best real-time performance, the detection accuracy is significantly lower than that of our method. In terms of comprehensive performance, HBMLCM is close to our methods. However, the ROC curve in Figure 10a shows that the detection ability of our method is much better than HBMLCM for low thermal targets. Moreover, the simulation also provides a theoretical basis for hardware-in-loop (HIL) experiments.

### 4.3. HIL Experiment

The HIL experiment was used to test the effectiveness of our algorithm in the fuze. In order to simulate the outdoor environment, we use a turntable to fix the fuze prototype. Four infrared small targets were placed in different directions in the field of view. The detection distance was set to 5 m according to the same equivalent distance between mortar and target. Moreover, considering the influence of the field of view, the diameter of the target placement range was 1.5 m, calculated by the detection distance and the parameters of the infrared imager.

**Figure 11:**
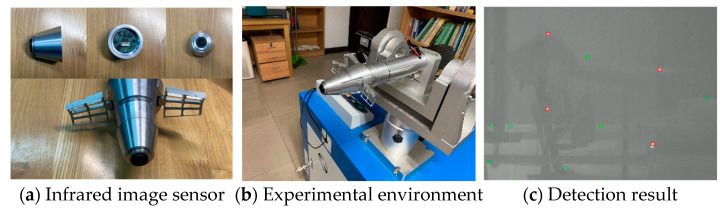
Experiment of HIL.

Figure 11a shows the prototype of the infrared image sensor and its strapdown design with the correction fuze. Figure 11b shows the fuze prototype fixed on the 3-DOF turntable. First, the experimental system was powered on. The turntable simulates the rotation of the mortar around the projectile’s axis at a speed of 2 r/s. At the same time, the fuze was turned on to keep the field of view stable. In addition, the random swing within 5 degrees of pitch and yaw direction was added to simulate the aerodynamics. The detection result of one frame is shown in Figure 11c. Although the disturbance deformed the infrared target, the proposed method could detect all the targets successfully.

Through simulation and the HIL experiment, our method was proven to be robust and was also suitable for the trajectory correction fuze. In terms of subjective and objective evaluation, it could be concluded that the performance of our algorithm is satisfactory compared with other state-of-the-art algorithms for small infrared target detection.

## 5. Conclusions

In this paper, a simple and effective method for small infrared target detection was proposed for the trajectory correction fuze. The three challenges, namely long detection distance, small variable size and minimal thermal signature, were solved. The research has made a great contribution to the application of infrared cameras to intelligent fuzes.

First, the characteristics of the fuze and trajectory were analyzed. The infrared image sensor was selected by calculating the parameters. Second, the spatial-only target detection method was used. Inspired by filtering methods, a density-distance space was proposed to detect the candidate targets from the original infrared image. Finally, an adaptive pixel growth method was presented to select real targets from seeds (candidate targets) by suppressing various kind of clutter.

Three experiments proved the robustness, effectiveness and applicability of the method, respectively. First, the outdoor equivalent detection experiment verified the correctness of the parameters of the infrared image sensor. The challenge of long-distance detection was overcome. Second, the simulation proved that our method has superior anti-noise detection, multiple target detection and small various targets’ size detection capability. Meanwhile, compared with six state-of-the-art algorithms, by ROC curve and running time in 5 different sequences, our method showed perfect detection accuracy and acceptable time consumption. Compared with Top-Hat, our method had better detection accuracy, and the running time was 0.0151 s slower. The HBMLCM has the same comprehensive performance as our method. However, our method had better capabilities of clutter suppression and low thermal target detection. Although the average running time of our method was 0.0057, 0.0096, 0.0031 and 0.0006 s slower than MinLocalLoG, LS_SVM, HBMLCM and MPCM, the order of magnitude was the same. Therefore, the challenge of minimal thermal signature has been solved. Finally, the HIL experiment generated a perfect detection result by the application of our method to the correction fuze based on the infrared image sensor.

As for the future work, it should be considered that the size of the target will enlarge during flight of mortar, which means that the small target detection method could not be used. Therefore, it is necessary to detect the key parts of the infrared target with large areas through the deep learning method.

## Figures and Tables

**Table 1:** Parameters of mortar and infrared image sensor.

Trajectory Parameters		Infrared Imager	
Detection distance (m)	1500	Focal length (mm)	19
Launch angle (degree)	53	Pixel pitch (μm)	17
Pitch range (degree)	60.3–65.2	Fov (degree)	17 × 13
Time (s)	8	Array format	320 × 240
Initial velocity (m/s)	272	Spectral band (μm)	7.5–13.5

**Table 2:** Experimental conditions.

Experimental Conditions	Detection Distance (m)	Target Size (m)	Temperature Difference (K)
Real Conditions	1500	2.3	15
Equivalent Conditions	65	0.1	7

**Table 3:** Details of the six original images.

No.	Background	Target Number	Image Size
1	Sea Building	2	284 × 213
2	Ground	3	220 × 140
3	Cloud Sky	3	281 × 240
4	Ground	4	220 × 140
5	Cloud Sky	4	250 × 200
6	Ground	6	220 × 140

**Table 4:** Average running time for a frame, of seven algorithms in five sequences (Seq.).

	Top-Hat (s)	MinLocalLoG (s)	LS_SVM (s)	HBMLCM (s)	RLCM (s)	MPCM (s)	Proposed (s)
Seq.1	0.0058	0.0155	0.0115	0.0181	1.9756	0.0213	0.0214
Seq.2	0.0059	0.0160	0.0120	0.0184	1.9616	0.0221	0.0214
Seq.3	0.0059	0.0154	0.0115	0.0179	1.9817	0.0213	0.0214
Seq.4	0.0061	0.0152	0.0113	0.0177	1.9799	0.0214	0.0211
Seq.5	0.0061	0.0149	0.0112	0.0178	1.9691	0.0217	0.0202
Average	0.0060	0.0154	0.0115	0.0180	1.9735	0.0216	0.0211

## Data Availability

Not applicable.
